# Role of estrogen receptors and Src signaling in mechanisms of bone metastasis by estrogen receptor positive breast cancers

**DOI:** 10.1186/s12967-017-1192-x

**Published:** 2017-05-04

**Authors:** Jen-Hwey Chiu, Che-Sheng Wen, Jir-You Wang, Chih-Yi Hsu, Yi-Fang Tsai, Shih-Chieh Hung, Ling-Ming Tseng, Yi-Ming Shyr

**Affiliations:** 1 Comprehensive Breast Health Center & Division of General Surgery, Department of Surgery, Taipei Veterans General Hospital, Taipei, Taiwan, ROC; 2 Division of General Surgery, Department of Surgery, Cheng-Hsin General Hospital, Taipei, Taiwan, ROC; 3 Institute of Traditional Medicine, School of Medicine, National Yang-Ming University, Taipei, Taiwan, ROC; 4 Department of Orthopedics, Cheng-Hsin General Hospital, Taipei, Taiwan, ROC; 5 Department of Orthopedics, Taipei Veterans General Hospital, Taipei, Taiwan, ROC; 6 Department of Pathology and Laboratory Medicine, Taipei Veterans General Hospital, Taipei, Taiwan, ROC; 7 School of Medicine, National Yang-Ming University, Taipei, Taiwan, ROC; 8 Institute of Clinical Medicine, School of Medicine, National Yang-Ming University, Taipei, Taiwan, ROC; 9 Integrative Stem Cell Center, China Medical University Hospital, Taichung, Taiwan, ROC; 10 Department of Surgery, Faculty of Medicine, School of Medicine, National Yang-Ming University, Taipei, Taiwan, ROC

**Keywords:** Estrogen receptor, Breast cancer, Snail, Bone metastasis, p190 RhoGAP

## Abstract

**Background/aim:**

Evidence shows that Luminal A breast cancer is likely to undergo bone metastasis, but the mechanisms involved remain unknown. This study’s aim was to demonstrate a correlation between estrogen receptor (ER) positivity and bone metastasis as the clinically preferred site of metastasis, as well as investigating the role of ERα-Src signaling in MCF-7 cells using Snail over-expression as an in vivo bone metastasis model.

**Methods:**

Clinically, the records of breast cancer with distant metastasis were retrospectively reviewed to correlate breast cancer subtypes and preferential metastatic sites. An in vivo bone metastasis model was created by injection of MCF-7 cells with/without Snail over-expression into the tibia of nude mice. The human MCF-7 cells that over-expressed (o/e) Snail were examined and the expression of epithelial–mesenchymal transitions (EMT) markers, ER-Src signaling proteins and p190 RhoGAP analyzed by Western blotting and real-time PCR. The role of ERα was elucidated using *ESR1* silence by transfecting shRNA (∆*ESR1*) into MCF-7 o/e Snail cells in vitro and in vivo.

**Results:**

The clinical results showed that ER ≥1% breast cancers showed a positive correlation with bone metastasis, which was found to be the preferred site of metastasis. An in vivo bone metastasis was successfully established using injection of MCF-7 o/e Snail cells into the tibia of nude mice, but no such metastasis was found using control MCF-7 cells. The proteins expressed in MCF-7 o/e Snail cells showed an EMT pattern, while those of the MCF-7 o/e Snail metastatic tissue showed a mesenchymal–epithelial pattern. There was an increase in cytosolic Src, p190 RhoGAP and nuclear ERα proteins, but not in Snail, in MCF-7 o/e Snail tissue compared to the same cell line in vitro. *ESR1* knock down decreased Src and p190 RhoGAP expression in vitro and also decreased the incidence of bone metastasis in vivo.

**Conclusion:**

We conclude that ER-Src signaling plays an important role in ER (+) breast cancer, which shows a high potential for bone metastasis.

**Electronic supplementary material:**

The online version of this article (doi:10.1186/s12967-017-1192-x) contains supplementary material, which is available to authorized users.

## Background

Breast cancer is the most common invasive female cancer worldwide and this is also true for Taiwan [1, 2]. Rapid advances made using genomic approaches, molecular analysis using immunohistochemistry, measurement of proliferative capacity, and gene expression profiles have allowed the further categorization of breast cancers into four types; these are the luminal A, luminal B, HER2 and basal-like subtypes [3–6]. Biological subtyping not only helps to provides feasible treatment strategies that correlate well with clinical outcome, but also helps to predicts whether the primary tumor has a preferential distant metastasis site [7].

The epithelial–mesenchymal transition (EMT), which is characterized by a loss of cell–cell adhesion, epithelial polarity, and the gain of mesenchymal properties, such as migration and invasiveness, is mechanism well known to be involved in tumor metastasis and invasion [8, 9]. Among many EMT regulators, which include Slug, ZEB, E47, Snail and TWIST1, over-expression of the latter two correlates well with tumor recurrence and a poor prognosis for breast cancer [10–13]. Recently, breast cancer subtypes have been identified as having a propensity to give rise to their first distant metastases at certain preferred body sites. For examples, luminal A breast cancers have a tendency to give rise first to bone metastases, while basal type cancers metastasize to liver and brain and HER2-enriched cancers give rise to liver and lung metastases [7]. Moreover, a recent investigation has suggested that mutational activation of ESR1 plays an important role in acquired endocrine resistance during breast cancer therapy [14]. However, the role of the estrogen receptor (ER) in the preferential metastasizing of Luminal A breast cancer to bone remains unexplored.

The Src family is a group of nonreceptor tyrosine kinases. Accumulating evidence has suggested that Src plays an important role in bone resorption and osteoclast activation, which seems to lead to the preferential bone metastasis of prostate cancer and the occurrence of late-onset bone metastasis in breast cancer [15, 16]. Recent clinical studies have been carried out to evaluate the efficacy of Src inhibitors in the treatment of solid tumors and breast cancer with bone metastasis [17, 18]. Nevertheless, the role of ER-Src signaling during Snail over-expression bone metastasis remains to be elucidated.

RhoGTPases and GTPase activating proteins (GAPs) contain a highly conserved GTPase activating domain that targets small GTPases; these latter proteins regulate intracellular cytoskeletal (actomyosin and microtubules) dynamics via their down-stream Rho kinase [19]. Among the many RhoGAPs, p190RhoGAP (p190) is a protein that has critical phosphorylation and protein interaction motifs in its middle domain [20]. A previous study has shown that p190 plays an important role in regulating cytoskeleton dynamics via inactivation of Rho signaling [21]. In addition, Src phosphorylates p190 at tyrosine 1105 (Y1105) allows enhancement of p190’s RhoGAP activity and Rho inactivation [22]. However, information on the relationship between the ER-Src-p190 RhoGAP axis and bone metastasis of breast cancer is not available.

Clinically, it was observed during our daily practice that breast cancer with a luminal A subtype would seem to have a significant incidence of bone recurrence as the first distant metastatic site. Experimentally, animal studies have shown that MCF-7 cells that are over-expressing Snail (MCF-7 o/e Snail) have up-regulated expression of *Snail*, *N*-*cadherin* and *Vimentin* and down-regulated expression of *E*-*cadherin* compared to MCF-7 cells, suggesting an EMT process is occurring. In contrast, MCF-7 o/e Snail cells in bone tissue have up-regulated *E*-*cadherin*, but down-regulated *Snail*, *N*-*cadherin* and *Vimentin.* These findings suggest in the latter case a mesenchymal–epithelial transition (MET) process is occurring. Ingenuity pathway analysis (IPA) of the differential gene expression between MCF-7 cells, MCF-7 o/e Snail cells and MCF-7 o/e Snail bone metastasis tissue demonstrates that ERα played a central role in the bone metastasis process (Additional file 1).

Bases on the above, we propose a hypothesis whereby ERα modulates Snail-regulated bone metastasis via the Src-p190 signaling pathway. The aim of this study was to demonstrate the correlation between ER (+) status and bone metastasis as the preferred site clinically and to investigate the role of ERα-Src signaling in MCF-7 o/e Snail cells using an in vivo bone metastasis model.

## Methods

### Subjects

Between Jan. 2004 and Dec. 2008, 1701 patients with breast cancer were admitted to Taipei Veterans General Hospital. Under the approval of Institutional Review Board (# 2014-11-001AC) of this hospital, their records were retrospectively reviewed in order to investigate the correlation between their sites of distant metastasis and various factors such as the expression of receptors (ER, PR) and their HER2/neu status. The first metastatic site of these patients was defined as metastasis involving a single organ, such as bone, lung, liver, brain and so on. Those who were firstly diagnosed as having multiple organs metastasis (≥2) were excluded. The mean follow up time was >60 months. The positivity of receptor status was defined as positive when ER or PR was ≥10% while, on the other hand, those having ER or PR <10% were classed as negative. The positivity of HER2/neu was defined as positive when staining was +++ by immunohistochemistry or amplified gene expression by FISH examination.

### Cell lines and reagents

The human breast cancer cell line MCF-7 (ER+, HER2-low) was obtained from the Food Industry Research and Development Institute (Taiwan, R.O.C.), while the Snail-over-expressed MCF-7 (MCF-7 o/e Snail) line obtained from Professor Hong SC, Department of Orthopedics, Taipei Veterans General Hospital. Both cell lines were routinely screened to show they were free from mycoplasma contamination. They were maintained in DMEM supplemented with 10% fetal bovine serum (FBS), 2 mM l glutamine, 1.5 g/L NaHCO_3_, 0.1 mM NEAA, 1.0 mM sodium pyruvate, penicillin/streptomycin (Invitrogen, NY, USA) and 10% fetal calf serum (Chemicon, CA, USA).

### Western blotting analysis

Cultured cells were lysed in a buffer containing 150 mM KCl, 10 mM Tris pH 7.4 and 1% Triton X-100 together with phosphatase inhibitor and protease inhibitors cocktail (Complete Mini; Roche, Mannheim, Germany). The protein concentrations of the cell homogenates were measured using the Bradford method [23]. Thirty microgram of proteins were separated using 10% SDS-PAGE and then transferred to a nitrocellulose membrane (Hybond-C; Amersham Biosciences, NJ, USA). The membrane was blocked with 5% bovine serum albumin, which was followed by probing with various specific antibodies such as Src antibody, C-term (GTX61220, GeneTex, Texas, USA), p190-B RhoGAP antibody (GTX61259, GeneTex, Texas, USA), ERα (GTX100634, GeneTex, San Antonio, Texas), anti-α-tubulin 1A (GTX109832, GeneTex, San Antonio, Texas), and anti-β-actin (GTX109639, GeneTex, San Antonio, Texas), N-cadherin (C-terminus clone EPR1792Y. #04-1126 Merck Millipore, Billerica, Massachusetts), E-Cadherin E (#3195) and Vimentin (GTX100619, GeneTex, Texas, USA) and Snail (#AP2054a, ABGENT, San Diego, CA) antibodies. These were purchased commercially (Cell signaling, Danvers, Massachusetts).

### Total RNA extraction and reverse transcription-PCR

Total RNA was isolated using a modified single-step guanidinium thiocyanate method [24] (TRI REAGENT, T-9424, Sigma Chem. Co., St. Louis, MO, USA). Complementary DNA (cDNA) was prepared from the total RNA using a First Strand cDNA Synthesis Kit (Invitrogen, CA, USA). Changes in de novo gene synthesis in each treatment group was detected by reverse transcriptase-polymerase chain reaction (RT-PCR). The gene expression of p190 RhoGAP, Src, and SNAIL were elucidated using commercially available primers. The primers for GAPDH were Forward-5′-GGAGCGAGATCCCTCCAAAAT-3′, Reverse-5′-GGCTGTTGTCATACTTCTCATGG-3′; for Src were Forward, 5′-GAACCCGAGAGGGACCTTC-3′, Reverse-5′-GAGGCAGTAGGCACCTTTTGT-3′; for E-cadherin were, Forward-5′-CGAGAGCTACACGTTCACGG-3′, Reverse-5′-GGGTGTCGAGGGAAAAATAGG-3′; for N-Cadherin were Forward-5′-CAACTTGCCAGAAAACTCCAGG-3′, Reverse-5′-ATGAAACCGGGCTATCTGCTC-3′; for p190 RhoGAP were Forward-5′-GCACAACTCGACCTTCTTTGG-3′, Reverse-5′-CGAAATAGAGTATGTCGGCCTTG-3′; for ESR1 were Forward-5′-GCACCCTGAAGTCTCTGGAA-3′ Reverse-5′-TGGCTAAAGTGGTGCATGAT-3′; and for hSnail1 were Forward-5′-GCTCCACAAGCACCAAGAGT-3′ Reverse-5′-ATTCCATGGCAGTGAGAAGG-3‘.

Any possible contamination of the various PCR components was excluded by performing a PCR reaction with these components in the absence of RT product for each set of experiments (non-template control, NTC). Quantification of the RNA transcripts was carried out and the relative mRNA expression levels of the various specific genes were normalized against the expression level of *GAPD* in the same RNA extract. All samples were analyzed in triplication.

### Short hairpin RNA (shRNA) transfection

Short hairpin RNA (shRNA) was used to silence the *ESR1* gene and the shRNA was obtained from Academia Sinica. One day after the Snail-over-expressing MCF-7 (MCF-7 o/e Snail) cell line was subcultured, the cells (30–40% confluent) were transfected for 24 h with shRNA against *ESR1* or a non-silencing control; this was carried using GenePORTER 2 transfection reagent (Genlantis, San Diego, CA, USA) dissolved in Optimem (Invitrogen) at a final concentration of 80 nM. Next the MCF-7 o/e Snail cells were allowed to recover before further experiments were carried out. After several passages, a ∆*ESR1* o/e Snail MCF-7 cell line was established by puromycin selection. The transfection efficiency was validated by Western blot analysis.

### In vivo bone metastasis-like model

An in vivo bone metastasis model was established using either a tumor cell xenograft or intratibial injection of cancer cells. Study protocols, which involved experiments using mice, were approved (#1041118) by the Institutional Animal Committee of Yang-Ming University. All animals were treated under the regulations of the “Guide for the care and use of laboratory animals” [DHHS publication No. (NIH), revised 1996] and the “Improving bioscience research reporting for animal research”. Immunodeficient NU-Foxn1nu mice were obtained from National Laboratory Animal Center (Taipei, Taiwan, ROC). They were given ad libitum access to food and water and were maintained in a specific pathogen-free environment with 12 h light–dark cycle at 22–24 °C and 50% humidity under the regulations of animal care committee of National Yang-Ming University. The mice were used for the experiments at 8 weeks of age. MCF-7 o/e Snail cells and MCF-7 o/e Snail knocked down ESR1 (MCF-7 o/e Snail, ∆ESR1) cells were injected into the right tibia via the knee joint. In total 1 × 10^7^ cells/0.1 mL PBS were used for each mouse, and this gave rise to a noticeable solid tumor around the injection site at day 14. The left site of the same mouse was used as the negative control [25, 26]. After the tumor had formed, the mice were sacrificed when the tumor size was less than 2% of body weight and tissue samples were then collected for further analysis.

### Statistic analysis

Correlation between ER positivity and metastatic site was analyzed using Fisher’s exact test. Experimental results are expressed as the mean ± SEM. Differences between two groups were analyzed by student t test or Mann–Whitney U test while those between groups at each time point were identified by one-way ANOVA, followed by Dunnett’s post hoc test. A *p* value of <0.05 is considered statistically significant compared to vehicle or no treatment group.

## Results

### Correlation of bone metastasis as the first distant metastatic site with the ER (+)/PR (+), and HER2 (−) breast cancer subtypes

Between Jan. 2004 and Dec. 2008, 1701 patients with breast cancer were admitted to Taipei Veterans General Hospital, and their records were reviewed. Among them, 122 patients with distant metastasis had full records regarding the expression level of estrogen receptor (ER), while 120 patients had similar records for the progesterone receptor (PR). There was a positive correlation between ER ≥10% (*p* = 0.002) and PR ≥10% (*p* = 0.003), either combination with HER2 expression (*p* = 0.027) and bone as the organ of first distant metastasis (Table 1).

**Table 1:** Correlation of bone metastasis and breast cancer subtypes

1st metastatic site	Bone	Other sites	*p* value
ER ≥ 10%	43	38	0.002*
ER < 10%	11	33	
PR ≥ 10%	34	25	0.003*
PR < 10%	19	43	
ER ≥ 10% or HER2 (+)	47	50	0.027*
ER < 10% and HER2 (−)	7	21	

*ER* estrogen receptor, *PR* progesterone receptor, *HER2* human epidermal growth factor receptor 2. The positivity of the receptors was defined in “Methods”. * p value analyzed by Chi Square test. Other sites included lung, liver, brain and visceral organs

### Expression of EMT markers in MCF-7 o/e Snail cells

**Fig. 1:**
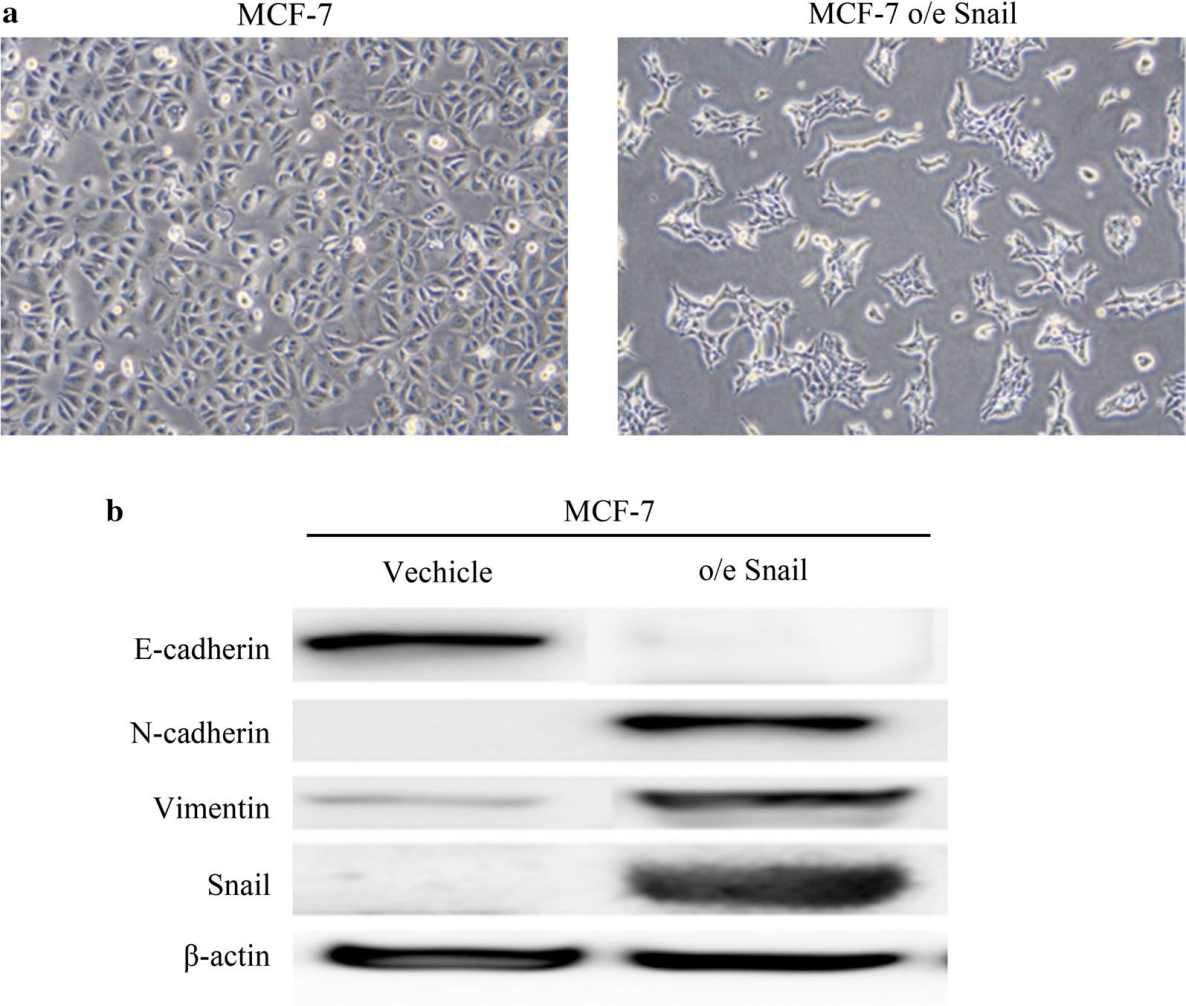
Expression of epithelial–mesenchymal transition (EMT) markers in MCF-7 o/e Snail cells. Using MCF-7 and MCF-7 o/e Snail cell lines as in vitro model (**a**), Expressions of EMT markers, such as E-cadherin, N-cadherin, Vimentin and Snail were analyzed by Western blot (**b**)

We used MCF-7 and MCF-7 o/e Snail cell lines as an in vitro model (Fig. 1a). The MCF-7 o/e Snail cells were found to show a typical expression profile of EMT markers, namely increased expression of N-cadherin, Vimentin and Snail and decreased expression of E-cadherin compared to the MCF-7 cell control (Fig. 1b).

### The in vivo bone metastasis-like model

**Fig. 2:**
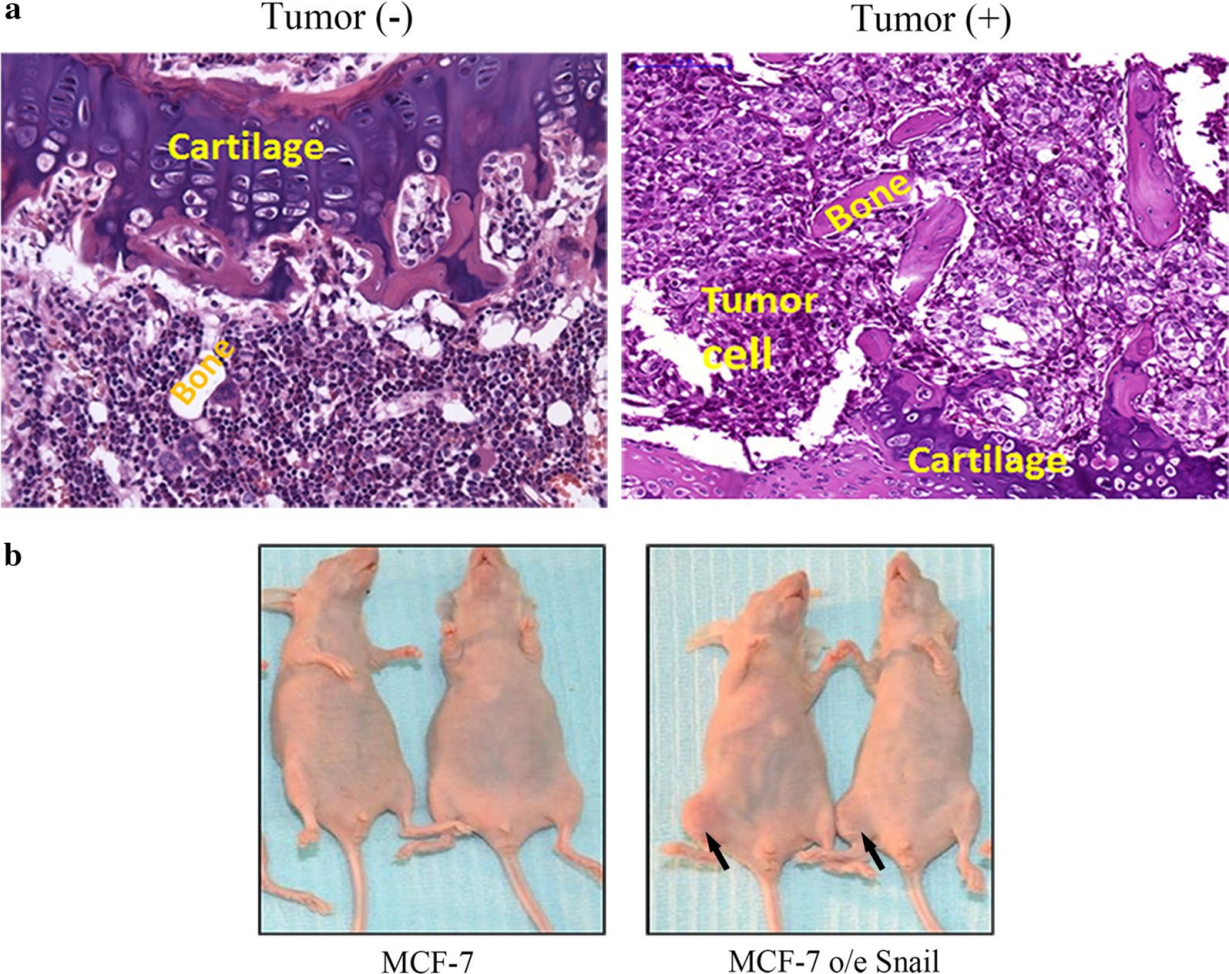
In vivo bone metastasis-like model. Bone metastasis-like model (bone metastasis) was established by intra-tibia injection of MCF-7 o/e Snail, but not MCF-7 wild type (MCF-7), (**a**, **b**). There was a significantly increased incidence of tumor formation in MCF-7 e/e Snail group than MCF-7 wild type (Table 2)

An in vivo bone metastasis-like model (bone metastasis) was established using intratibial injection of MCF-7 o/e Snail; as a control the MCF-7 wild type (MCF-7) was used in a similar manner, but in this no tumors developed, (Fig. 2a, b). Thus there was a significantly increased incidence in tumor formation within the MCF-7 e/e Snail group compared to the MCF-7 wild type group (Table 2).

**Table 2:** Incidence of tumor formation after intratibial injection of MCF-7 over-expressed Snail

	Tumor (+)	Tumor (−)	p value
MCF-7	0	6	0.001*
MCF-7 o/e Snail	23	8	

MCF-7 o/e Snail, MCF-7 over-expressed Snail protein. Intratibial injection of cancer cells were described in “Methods”

* p value analyzed by Fisher exact test

### Differential expression of EMT markers in MCF-7 o/e Snail cells and MCF-F o/e Snail tissue

**Fig. 3:**
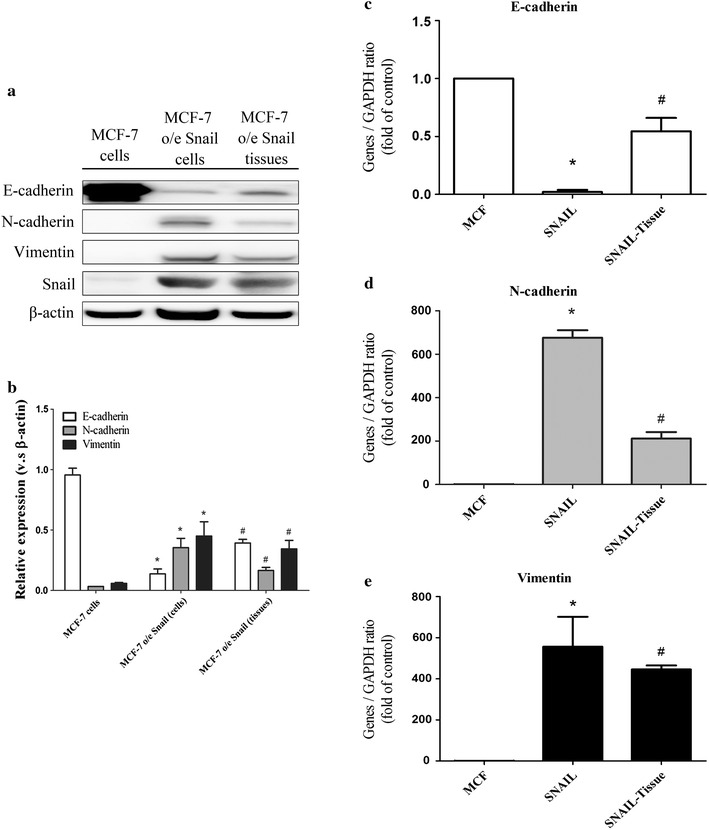
Differential expression of epithelial–mesenchymal transition (EMT) markers in MCF-7 o/e Snail cells and MCF-F o/e Snail tissues. Proteins expressions of EMT markers of MCF-7 cells, MCF-7 o/e Snail cells and those obtained from tumor tissue via intratibial injection of MCF-7 o/e cells, denoted as MCF-7 o/e Snail tissues were analyzed with Western blot (**a**) and quantified (**b**). The transcripts encoding these proteins such as E-cadherin (**c**), N-cadherin (**d**), and Vimentin (**e**) were analyzed with real-time PCR. *p < 0.05 vs MCF-7 cells by student t test; ^#^p < 0.05 vs MCF-7 o/e Snail (cells) by student t test

When comparing the proteins expressed by MCF-7 o/e Snail cells with those expressed by the tumor tissue created by intra-tibia injection of MCF-7 o/e cells, which is denoted as MCF-7 o/e Snail tissue from hereon, it was found that the former showed an EMT expression pattern, namely, increased N-cadherin, Vimentin and Snail and decreased E-cadherin expression, while the latter showed opposite reciprocal changes in these proteins, namely a MET pattern, either at protein level (Fig. 3a, b) or at transcriptional level (Fig. 3c–e).

### Expression of Src, p190 RhoGAP, and ERα in the MCF-7 o/e Snail cells and MCF-F o/e Snail tissue

**Fig. 4:**
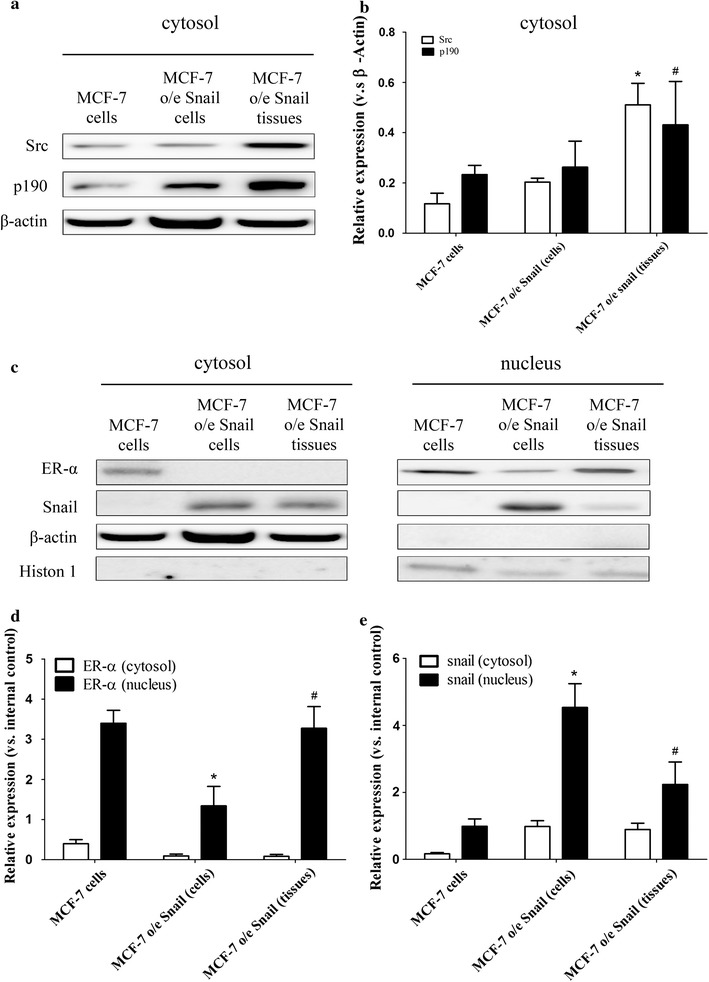
Expression of Src, p190 RhoGAP, and ERα in MCF-7 o/e Snail cells and MCF-F o/e Snail tissues. The proteins in MCF-7 cells, MCF-7 o/e Snail cells and MCF-F o/e Snail tissues were lyzed as described in “Methods”. The cytosolic expressions of Src, p190 RhoGAP were analyzed by Western blot (**a**) and quantified (**b**). *p < 0.05 vs MCF-7 cells by Mann–Whitney U test; ^#^p < 0.05 vs MCF-7 cells, by student t test. The nuclear expressions of Snail and ERα (**c**, **d**, **e**) were quantified. *p < 0.05 vs MCF-7 cells; ^#^p < 0.05 vs MCF-7 o/e Snail (cells) by Mann–Whitney U test

The expression of Src, p190 RhoGAP and Erα were compared between MCF-7, MCF-7 o/e Snail cells and MCF-7 o/e Snail tissue by Western blotting. In MCF-7 o/e Snail cells compared to MCF-7 wild type cells, there was no significant change in cytosolic Src protein expression, but there was a trend towards increased p190 RhoGAP expression. This contrasted with the situation with MCF-7 o/e Snail tissue, where there was a marked increase in cytosolic Src and p190 RhoGAP expression (Fig. 4a, b). Protein expression in MCF-7 o/e Snail cells was compared with protein expression in MCF-7 o/e Snail tissue and it was found that the former one had a nuclear pattern of increased Snail and decreased ERα expression, while the latter one had reciprocal changes in these proteins, namely decreased Snail and increased ERα expression in the nucleus (Fig. 4c–e).

### ERα modulated Src and p190 RhoGAP gene expression MCF-7 o/e Snail cells

**Fig. 5:**
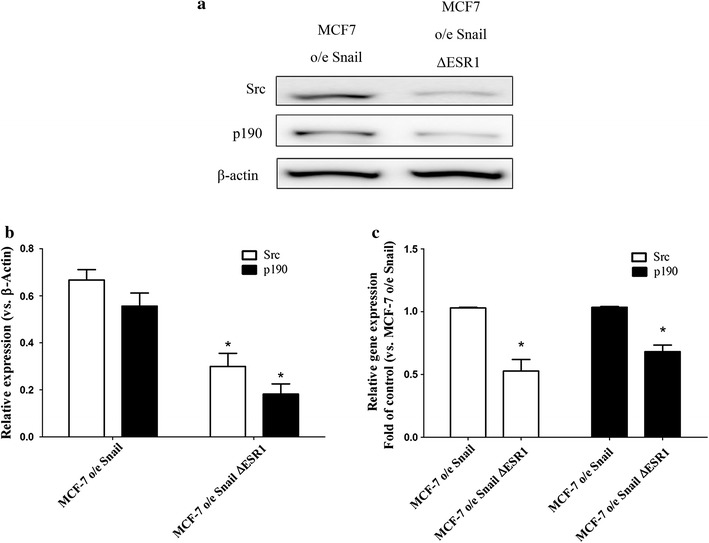
ERα modulated Src and p190 RhoGAP gene expression MCF-7 o/e Snail cells. ERα gene expression was knocked down (MCF-7 o/e Snail ∆ESR1) by transfection of short hairpin RNA on MCF-7 o/e Snail cells. The expression of Src and p190 RhoGAP, either on protein level (**a**, **b**), or on mRNA level (**c**). *p < 0.05 vs MCF-7 o/e Snail (cells) by Mann–Whitney U test

To elucidate the role of ERα on Src and p190 RhoGAP, ERα gene expression was knocked down (MCF-7 o/e Snail ∆ESR1) by transfection of MCF-7 o/e Snail cells with short hairpin RNA. The results showed that MCF-7 o/e Snail ∆ESR1 cells had significantly lower levels of Src and p190 RhoGAP gene expression, both at the protein level (Fig. 5a, b) and the mRNA level (Fig. 5c). These results suggest that ERα modulates Src and p190 RhoGAP gene expression in MCF-7 o/e Snail cells.

### The role of ERα on in vivo bone metastasis

To test the hypothesis that ERα plays a key role in ER (+) breast cancer and its relationship with bone metastasis, in vivo bone metastases were created by injection of MCF-7 o/e Snail with/without *ESR1* knock down. The results demonstrated that *ESR1* knock down of MCF-7 o/e Snail cells decreased the incidence of bone metastasis in vivo (*p* = 0.0217, Fisher exact test, Table 3).

**Table 3:** Incidence of tumor formation after intratibial injection of MCF-7 over-expressed Snail and knocked down ESR1

	Tumor (+)	Tumor (−)	p value
MCF-7 o/e Snail	23	8	0.014*
MCF-7 o/e Snail ∆*ESR1*	1	5	

MCF-7 o/e Snail, MCF-7 over-expressed Snail protein. MCF-7 o/e Snail, ∆*ESR1*, MCF-7 over-expressed Snail protein and *ESR1* gene knocked down simultaneously. Intratibial injection of cancer cells were described in “Methods”

* p value analyzed by Fisher exact test

## Discussion

In this study, we demonstrated that there is a correlation between an ER (+) status and bone metastasis as the preferred metastatic site clinically. We then investigated the role of ERα-Src signaling in MCF-7 cells with Snail over-expression using a bone metastasis model in vivo. Although many clinical studies have shown a correlation between breast cancer subtypes and their patterns of distant metastasis [7, 27], we are the first to incorporate translational research by elucidating the role of ER-Src signaling in the mechanisms of bone metastasis, both in vitro and in vivo.

There is consensus that an experimental bone metastasis model is not the same situation as that observed in humans. Nonetheless, a bone metastasis-like model involving intratibial injection of cancer cells is generally accepted as a suitable in vivo model when studying the carcinogenesis process related to bone sarcoma [25], the mechanisms of bone metastasis [26, 28], and the efficacy evaluation of a therapeutic agent [29].

The epithelial–mesenchymal transition (EMT) and its reverse process (the mesenchymal–epithelial transition or MET) are critical during embryogenesis [30]. Among many EMT inducers, which include Snail1, Slug, Twist, FOXC2 etc., Snail is well known to bind to the promoter of E-cadherin and repress its transcription, leading to loss of E-cadherin expression, a fundamental event in the EMT [31]. A previous study has demonstrated that Snail over-expression represses the de novo synthesis of ER-alpha and does this via the interaction of Snail with regulatory sequences at the ESR1 locus; this leads to increased cell invasiveness [32]. Our results from the in vivo bone metastatic model, where tumors occurred after injection of MCF-7 o/e Snail cells, but after not injection of MCF-7 cells, suggests that the EMT is a critical event during the bone metastatic process. It should be noted that there are reciprocal changes in the various EMT markers, namely E-cadherin, N-cadherin, Vimentin, and Snail, between MCF-7 o/e Snail cells and MCF-7 o/e Snail tissue (bone tissue), leading us to speculate that a reverse EMT, that is a MET, occurs during seeding of the tumor cells into the bone. The complexity of the bone environment might result in a range of different ligand-receptor interaction. For example, it is known that the administration of 17 beta-estradiol (E2) decreases Slug expression and increases E-cadherin in ERα over-expressing ERα(−) cell lines [33]. In addition, recent investigations have suggested that mutations that activate ESR1 play an important role in acquired resistance to hormonal therapy [14] and the preference towards bone metastasis among breast cancer patients (ASCO, annual meeting, 2016). Our results are in agreement with the above-mentioned studies.

The proto-oncogene c-src, a non-receptor tyrosine kinase, has been demonstrated with its cooperating partners to play an important role in the development of many cancers [34]. Src activation is highly associated with bone metastasis by prostate cancer [35] and by late-onset bone metastasis from breast cancer. C-Src overexpression is an independent predictor of a poor outcome among breast cancer patients with bone metastasis [36] and is a potential therapeutic target for patients with secondary bone metastasis [37].

Recent evidence has demonstrated that Src plays an important role in the signaling and cross talk between growth-related signaling pathways such as the ER pathways [38] and that this can occurs in either a direct or an indirect manner [39]. Interestingly, using microarray and ingenuity pathway analysis (IPA), differential gene expression between MCF-7 cells, MCF-7 o/e Snail cells and MCF-7 o/e Snail bone metastasis tissue demonstrated that ERα has direct interaction with Src pathway (Additional file 1). Furthermore, Src is able to phosphorylate p190 at tyrosine 1105 (Y1105), which has been shown to enhance p190’s RhoGAP activity and thus Rho inactivation [22]; this in turn leads to an increase in proliferative potential and a decrease in migratory activity, which is subsequently followed by colonization of bone tissue. Our results from the knock down of ESR1 shows that there is down-regulation of the de novo synthesis of Src and p190 RhoGAP at both the protein and the transcriptional level; this finally results in a decrease in the incidence of bone metastasis. Thus the above findings help to explain the mechanisms by which breast cancer leads to bone metastasis.

## Conclusion

In summary, there is a positive correlation between ER (+) and bone metastasis as the preferred site of metastasis in human studies. ERα-Src-p190 RhoGAP signaling, when investigated both in vitro and in vivo, seems to play an important role in explaining the process by which bone metastasis develops from breast cancer.

## Additional file

**Additional file 1.** Additional information.

## Abbreviations

ER: estrogen receptor
EMT: epithelial–mesenchymal transition
MCF-7 o/e Snail: MCF-7 with Snail over-expression
MCF-7 o/e Snail, ∆ESR1: MCF-7 with Snail over-expression and ESR1 knocked down
MCF-7 o/e Snail tissue: MCF-7 with Snail over-expression in tissue
MET: mesenchymal–epithelial transition
p190 RhoGAP: p190 Rho GTPase-activating proteins
