# Supplementary material for: Role of estrogen receptors and Src signaling in mechanisms of bone metastasis by estrogen receptor positive breast cancers
Source: J Transl Med. 2017 May 4;15:97. doi: 10.1186/s12967-017-1192-x (PMC5418839; doi:10.1186/s12967-017-1192-x)

Additional information

1. Heat map and Ingenuity Pathway Analysis. Using microarray heat map (left) and ingenuity pathway analysis (IPA, right), differential gene expression between MCF-7 cells, MCF-7 o/e Snail cells and MCF-7 o/e Snail bone metastasis tissue demonstrated that ERα played a central role during bone metastasis process.


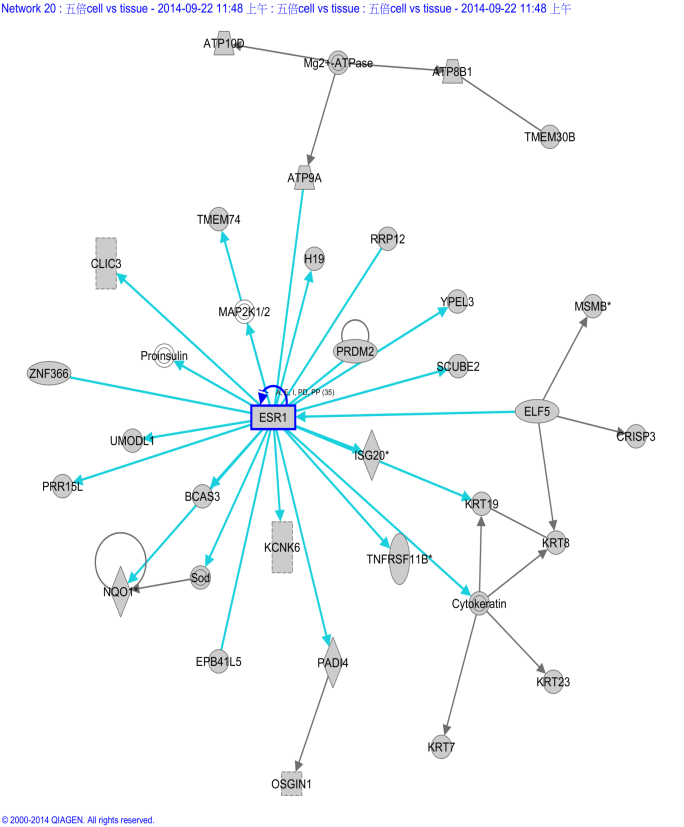


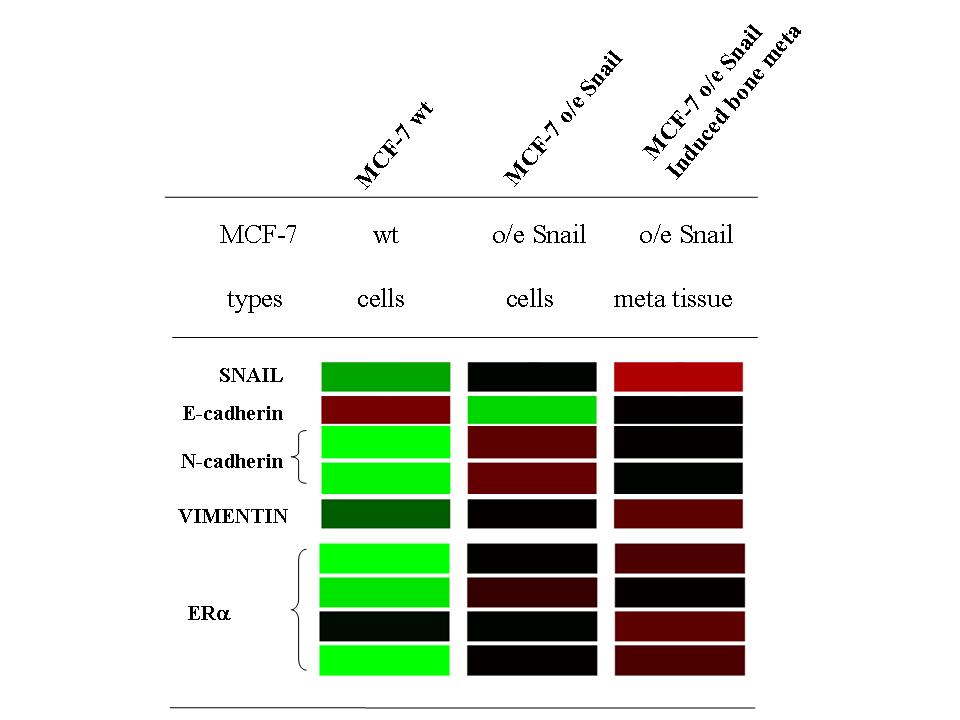


1. Ingenuity Pathway Analysis. Using ingenuity pathway analysis (IPA), differential gene expression between MCF-7 cells, MCF-7 o/e Snail cells and MCF-7 o/e Snail bone metastasis tissue demonstrated that ERα has direct interaction with Src signaling pathway during bone metastasis process.


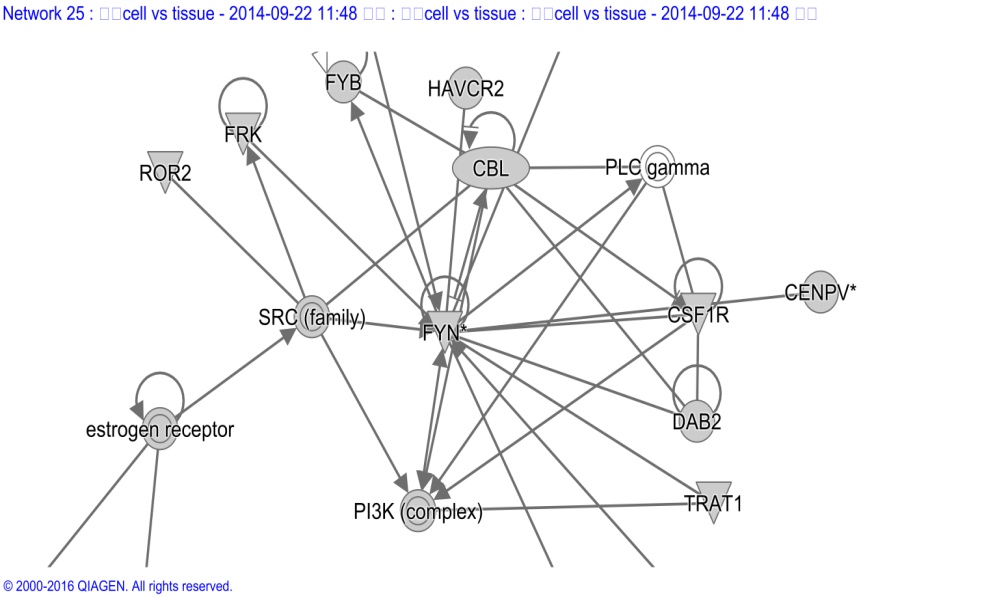

Supplement: Supplementary file 1 — Additional file 1. Additional information. [file 12967_2017_1192_MOESM1_ESM.docx]
